# National Heart Foundation of Australia: position statement on coronary artery calcium scoring for the primary prevention of cardiovascular disease in Australia

**DOI:** 10.5694/mja2.51039

**Published:** 2021-05-07

**Authors:** Garry LR Jennings, Ralph Audehm, Warrick Bishop, Clara K Chow, Siaw-Teng Liaw, Danny Liew, Sara M Linton

**Affiliations:** ^1^ University of Sydney Sydney NSW; ^2^ National Heart Foundation of Australia Melbourne VIC; ^3^ General Practice and Primary Health Care Academic Centre University of Melbourne Melbourne VIC; ^4^ Calvary Health Care Tasmania Lenah Valley Campus Hobart TAS; ^5^ Westmead Hospital Sydney NSW; ^6^ UNSW Sydney Sydney NSW; ^7^ Ingham Institute of Applied Medical Research Sydney NSW; ^8^ Monash University Melbourne VIC; ^9^ Royal Melbourne Hospital Melbourne VIC

**Keywords:** Coronary artery disease, Risk factors, Preventive medicine, Computed tomography, Cardiac imaging techniques

## Abstract

**Introduction:**

This position statement considers the evolving evidence on the use of coronary artery calcium scoring (CAC) for defining cardiovascular risk in the context of Australian practice and provides advice to health professionals regarding the use of CAC scoring in primary prevention of cardiovascular disease in Australia.

Main recommendations:

- CAC scoring could be considered for selected people with moderate absolute cardiovascular risk, as assessed by the National Vascular Disease Prevention Alliance (NVDPA) absolute cardiovascular risk algorithm, and for whom the findings are likely to influence the intensity of risk management. (GRADE evidence certainty: *Low*. GRADE recommendation strength: *Conditional*.)
- CAC scoring could be considered for selected people with low absolute cardiovascular risk, as assessed by the NVDPA absolute cardiovascular risk algorithm, and who have additional risk-enhancing factors that may result in the underestimation of risk. (GRADE evidence certainty: *Low*. GRADE recommendation strength: *Conditional*.)
- If CAC scoring is undertaken, a CAC score of 0 AU could reclassify a person to a low absolute cardiovascular risk status, with subsequent management to be informed by patient–clinician discussion and follow contemporary recommendations for low absolute cardiovascular risk. (GRADE evidence certainty: *Very low*. GRADE recommendation strength: *Conditional*.)
- If CAC scoring is undertaken, a CAC score > 99 AU or ≥ 75th percentile for age and sex could reclassify a person to a high absolute cardiovascular risk status, with subsequent management to be informed by patient–clinician discussion and follow contemporary recommendations for high absolute cardiovascular risk. (GRADE evidence certainty: *Very low*. GRADE recommendation strength: *Conditional*.)

**Changes in management as a result of this statement:**

CAC scoring can have a role in reclassification of absolute cardiovascular risk for selected patients in Australia, in conjunction with traditional absolute risk assessment and as part of a shared decision‐making approach that considers the preferences and values of individual patients.

Cardiovascular disease (CVD) is a leading cause of death in Australia^1^ and contributes to a significant health care burden.^2^ Australian guidelines for management of CVD risk in primary prevention recommend the assessment of absolute CVD risk, using the algorithm developed by the National Vascular Disease Prevention Alliance (NVDPA), which calculates an individual’s risk of a CVD event over a 5‐year period.^3^ Additional risk modifiers, such as coronary artery calcium (CAC) scoring, have been investigated as a way to improve the predictive performance of established risk assessment algorithms.^4^, ^5^, ^6^, ^7^, ^8^, ^9^, ^10^, ^11^, ^12^, ^13^, ^14^, ^15^, ^16^, ^17^, ^18^, ^19^, ^20^, ^21^, ^22^, ^23^

CAC scoring measures the amount of calcium in the coronary arteries from a computed tomography (CT) scan of the heart. A positive CAC score, measured in Agatston units (AU), is a marker of atherosclerosis, with increasing CAC scores correlating to increasing risk of CVD events and a CAC score of 0 AU indicating an absence of CAC and a low risk of CVD events.^24^, ^25^ Evidence for the ability of CAC scoring to improve the predictive performance of traditional risk assessment models, and for the ability of CAC‐guided management to reduce CVD morbidity and mortality, is evolving.

This position statement provides practical advice to health professionals regarding the use of CAC scoring to assist in defining risk in the primary prevention of CVD in Australia. It is not intended to be an update to Australian absolute CVD risk guidelines, nor is it our purpose to compare CAC scoring and absolute CVD risk equations as standalone predictors of risk. Rather, this article outlines a position specifically on the use of CAC scoring in the context of, and ancillary to, standard methods of risk assessment. The National Heart Foundation of Australia acknowledges that the limited availability of Australian published literature affects the certainty of the evidence for the role of CAC scoring in the Australian setting. However, we recognise the need for practical guidance for the use of CAC scoring, and thus these conditional recommendations with expert consensus are based on the Australian and international evidence available at the time. This position statement builds upon a previous consensus statement from the Cardiac Society of Australia and New Zealand.^26^

## Methods

Studies relevant to CAC scoring from a 2018 United States Preventive Services Task Force (USPSTF) systematic review of non‐traditional risk factors in CVD risk assessment^27^ were used as a foundation for this position statement. A literature search was conducted using methods adapted from the 2018 UPSTF systematic review to capture newly published studies. Reference lists from key consensus statements and guidelines were hand‐searched and surveillance of key journals was conducted through to June 2019, using the same inclusion criteria as the adapted search strategy (Supporting Information, appendix 1). Two additional studies were subsequently included for review. All studies were appraised for quality of evidence using the Grading of Recommendations Assessment, Development and Evaluation (GRADE) framework for improvement in CVD risk prediction and reduction in CVD events (Supporting Information, appendix 1).^28^, ^29^

The Heart Foundation appointed an expert reference group comprising a mix of health professionals who provided advice for evidence interpretation and formulation of recommendations. The recommendations were formed with consideration of the certainty of the evidence, the balance of benefits and harms, variability in patient values and preferences, and resource considerations. A draft of this article was open for a 30‐day period of public consultation in 2020 to capture stakeholder views and facilitate engagement. Governance processes were implemented to ensure transparency, minimise bias and manage conflict of interest during the development of this position statement.

## Recommendations

The Heart Foundation position is that CAC scoring can have a role in the reclassification of absolute CVD risk for selected, asymptomatic patients in Australia, in conjunction with traditional absolute CVD risk assessment and as part of a shared decision‐making approach that considers the preferences and values of individual patients. The conditional recommendations presented here (Box 1) reflect that this is a rapidly evolving area with emerging evidence, improved equipment limiting radiation exposure, and reducing out‐of‐pocket costs to consumers in the absence of public reimbursement. We also recognise that the comparator in our evidence review, risk assessment using the NVDPA algorithm alone, is not supported by randomised controlled trial data. However, it is difficult to directly compare the certainty of evidence for the NVDPA algorithm, as a different appraisal framework was used at the time of publication. While historically there have been many challenges associated with clinical trials for the use of CAC scoring,^30^ the results of novel clinical trials currently underway will likely have an impact on the certainty of evidence for CAC scoring.^31^ Lastly, we note that the recommendations presented in this article may change with forthcoming updates to the 2012 NVDPA absolute cardiovascular risk guidelines.

Box 1 **Recommendations for the use of coronary artery calcium (CAC) scoring in primary prevention of cardiovascular disease in Australia**

**Table:** 

CAC scoring could be considered for selected people with moderate absolute cardiovascular risk, as assessed by the NVDPA absolute cardiovascular risk algorithm, and for whom the findings are likely to influence the intensity of risk management. (GRADE evidence certainty: *Low*. GRADE recommendation strength: *Conditional*.) CAC scoring could be considered for selected people with low absolute cardiovascular risk, as assessed by the NVDPA absolute cardiovascular risk algorithm, and who have additional risk‐enhancing factors that may result in the underestimation of risk. (GRADE evidence certainty: *Low*. GRADE recommendation strength: *Conditional*.) If CAC scoring is undertaken, a CAC score of 0 AU could reclassify a person to a low absolute cardiovascular risk status; with subsequent management to be informed by patient–clinician discussion and follow contemporary recommendations for low absolute cardiovascular risk. (GRADE evidence certainty: *Very low*. GRADE recommendation strength: *Conditional*.) If CAC scoring is undertaken, a CAC score > 99 AU or ≥ 75th percentile for age and sex could reclassify a person to a high absolute cardiovascular risk status, with subsequent management to be informed by patient–clinician discussion and follow contemporary recommendations for high absolute cardiovascular risk. (GRADE evidence certainty: *Very low*. GRADE recommendation strength: *Conditional*.)

AU = Agatston units; GRADE = Grading of Recommendations Assessment, Development and Evaluation; NVDPA = National Vascular Disease Prevention Alliance.

### Evidence for recommendations

The full evidence review and evidence appraisal results can be found in the Supporting Information, appendix 1.

Several cohort studies have reported improvement in predictive performance with the addition of CAC scoring to the Framingham Risk Score and pooled cohort equation risk models, indicated by improvement in calibration, discrimination and reclassification (Box 2).^4^, ^5^, ^6^, ^7^, ^8^, ^9^, ^10^, ^11^, ^12^, ^13^, ^14^, ^15^, ^16^, ^17^, ^18^, ^19^, ^20^, ^21^, ^22^, ^23^ The most significant improvements in reclassification, indicated by improvement of the net reclassification index, were generally observed in subjects with intermediate 10‐year CVD risk (Supporting Information, appendix 1).^4^, ^5^, ^6^, ^7^, ^8^, ^9^, ^10^, ^11^, ^12^, ^13^, ^14^, ^15^, ^16^, ^17^, ^18^ Comparisons between these studies, and determining the clinical relevance of the findings, are challenging due to lack of summary statistic reporting in the studies and a lack of a consensus in the literature for the analysis of these measures.^27^, ^35^ In addition, there are limitations to the direct applicability of this evidence to Australian practice, as the comparator risk prediction models compute 10‐year risks of CVD whereas the NVDPA algorithm computes a 5‐year risk of CVD.

Box 2 **Terminology and measures used to assess the value of adding coronary artery calcium (CAC) scoring to existing prediction models**

**Table:** 

Terminology	Description
Calibration	Calibration refers to the agreement between observed and expected outcomes. The studies in our evidence review which reported on calibration used $\chi^{2}$ analyses (Supporting Information, appendix 1).
Discrimination	Discrimination refers to the ability of a risk prediction model to distinguish between individuals who will or will not have an event. This is measured by the C‐statistic, also referred to as the area under the receiver‐operator curve. It is the probability that for a pair of individuals, where one had an event and the other did not, the individual who experienced an event had a higher estimated probability according to the prediction model, and generally lies between 0.5 and 1.0.^32^ Improvements in discrimination for a new prediction model are assessed by the difference in C‐statistic compared with an old model. Guidance about the clinical meaning of changes in C‐statistic is limited, but the 2018 USPSTF review gives the following as a practical definition: a change of > 0.1 can be considered “large”, changes of 0.05–0.1 can be considered “moderate”, changes of 0.025 to < 0.05 can be considered “small”, and changes of < 0.025 can be considered “very small”.^27^
	In our evidence review, 19 studies (69 569 participants) reported improvements in discrimination when CAC scoring was added to FRS models compared with FRS alone. The change in C‐statistic ranged from 0.038 to 0.16, in favour of the models where CAC scoring was added to FRS models (Supporting Information, appendix 1).
Reclassification	Reclassification refers to the ability of a new risk model to appropriately reclassify subjects into the correct risk strata. The studies that reported on reclassification in our review have generally reported this improvement in terms of the NRI. The NRI is a measure of the net extent that a new model compared with an old model reclassifies the risk of individual subjects according to specified risk strata, considering both correct overestimating the risk and incorrect underestimating the risk of those who did have an event as well as correct underestimating the risk and incorrect overestimating the risk of those who did not have an event in a defined time period.^27^ It is not a simple proportion, rather it is a sum of proportions. There are some variations in how it is calculated which may limit its direct comparison across studies, and a common limitation noted is its tendency to overstate the incremental value of a new predictor.^33^, ^34^ Positive results indicate improvement in reclassification with the new model, and the maximum possible value for an NRI is 2.
	Assessment of prediction performance improvement is generally best done with examination of multiple measures, including the ones described here. Other common measures are the net benefit function, integrated discrimination statistic and the Brier score.
	In our evidence review, 15 studies (57 409 participants) reported an NRI for models that included CAC scoring in addition to FRS, compared with FRS alone. The NRI ranged from 0.11 to 0.55 overall, in favour of the models that included CAC scoring, with results ranging from 0.21 to 0.659 in subanalyses of intermediate risk subjects when performed (Supporting Information, appendix 1).

FRS = Framingham Risk Score; NRI = Net Reclassification Index; USPSTF = United States Preventive Services Task Force.

With respect to evidence of clinical benefit, two randomised clinical trials assessed the impact of CAC scoring on rates of CVD events in populations without known CVD. The St Francis Heart Study found no significant difference in a composite of CVD events between subjects who received atorvastatin 20 mg plus vitamin C plus vitamin E compared with placebo in subjects with a CAC score ≥ 80th percentile for age and sex.^36^ The Early Identification of Subclinical Atherosclerosis by Non‐invasive Imaging Research (EISNER) study found that use of CAC scoring in addition to the Framingham Risk Score calculation and risk factor counselling was associated with better CVD risk factor control after 4 years compared with subjects who only received the Framingham Risk Score calculation and risk factor counselling.^37^ The study also measured rates of myocardial infarction and cardiac mortality as a secondary outcome, finding no significant difference between patients who did and did not undertake CAC scoring. The certainty of these findings in relation to evidence of clinical benefit is limited, as neither study was powered to detect differences in morbidity and mortality.

A recent retrospective cohort analysis of a large CAC score registry investigated the association of CAC scores with benefit from statin therapy.^38^ This study found that in patients with a CAC score greater than 0 AU, statin therapy was associated with a reduced risk of major adverse cardiovascular event compared with no statin therapy. There was no significant difference in major adverse cardiovascular event risk with statin therapy compared with no statin therapy for patients with a CAC score of 0 AU.

Determining CAC thresholds for reclassification of risk is challenging in the absence of Australian data for CAC‐guided CVD risk management. US guidelines outline CAC score thresholds and subsequent reclassification of risk status for individuals initially assessed as borderline and intermediate atherosclerotic CVD risk (10‐year risk range, 5–20%).^39^, ^40^, ^41^ The rationale for these thresholds is based on the atherosclerotic CVD risk associated with specific CAC scores, in comparison to the CVD risk for which clinical benefits from more intensive risk modification therapies have been demonstrated. There were assumptions involved in applying the rationale for these thresholds to Australian clinical practice, as the NVPDA algorithm computes a 5‐year CVD risk. However, in the absence of Australian data, there was no alternative to using the CAC score thresholds from the US guidelines to reclassify the 5‐year risk categories as computed by the NVPDA algorithm if CAC is measured.

### Benefits and harms

The conditional status of our recommendations reflect possible benefits in CVD‐related morbidity and mortality that are yet to be proven in randomised controlled clinical trials. At this stage, the benefits are observed for clinical decision making and the potential for CAC scoring to refine risk assessment. A recent analysis of about 1000 asymptomatic individuals with family history of early onset coronary artery disease demonstrated the potential of CAC scoring to reclassify risk in an Australian population.^42^ In this study, 19% of patients deemed at moderate risk using the Australian risk algorithm had CAC scores of 0 AU (14 out of 75 subjects). Of the subjects initially deemed at low risk using the Australian algorithm, 77% had CAC scores above 100 AU (116 out of 151 patients).

The benefit of refined risk assessment is that more individuals can receive risk management interventions of an intensity appropriate for their level of absolute cardiovascular risk. We posit that a CAC score of 0 AU could also reduce anxiety for individuals, and possibly also their clinicians, who are reluctant or unable to tolerate more intensive risk management therapies otherwise indicated by their initial absolute risk assessment from the NVDPA algorithm.

Radiation exposure during CAC measurement is another important consideration. A systematic review reported the dose of radiation used in CAC measurement to range from 1 mSv to 2 mSv.^27^ There are anecdotal reports that newer equipment used in some centres in Australia use much lower radiation doses. The radiation doses from CT imaging for the purpose of CAC scoring are generally considered to align with published guidelines for radiation dose minimisation.^43^, ^44^, ^45^

The potential for incidental findings from CT imaging may also add to anxiety and incur additional follow‐up costs.^46^ However, incidental findings that lead to early diagnosis and treatment of comorbidities may represent a benefit for some patients. Incidental findings from CAC measurement should be managed on their merits according to best‐practice recommendations for follow‐up of incidental findings on radiology.

The potential for unnecessary downstream medical testing as a result of a high risk CAC result is also an important consideration. The evidence for this occurring in Australian practice is limited at the time of writing. The EISNER study found that overall downstream medical testing and costs were not significantly different between subjects that received CAC scoring and those who did not, balanced by lower and higher resource utilisation for subjects with a CAC score of 0 AU and ≥ 400 AU respectively.^37^ Subsequent risk management, including further medical testing, should follow contemporary guidelines for an individual’s risk category, regardless of whether a CAC score has been used in the assessment of absolute cardiovascular risk.

### Resource considerations

The cost of CAC scoring should be included in discussions of benefits and harms with patients when considering this test. At the time of writing, CAC scoring is not publicly funded in Australia and the cost lies with individual patients. The cost can vary considerably depending on the provider, and potentially limits accessibility to some segments of the community. The potential impact of the CAC scoring cost on health equity was an important factor in our decision to make our recommendations conditional, rather than routine. We note that there have been no Australian cost‐effectiveness data published to date. International cost‐effectiveness data for CAC scoring cannot be directly applied to its use in the Australian population due to significant variation in methods and health care systems.

It is recognised that technology is improving and contemporary high quality imagers have higher sensitivity and lower radiation doses than equipment used in some of the published literature. Although different standards no doubt apply in the acquisition and analysis of scans at centres around Australia, this issue is out of the scope of the present review. Published data are considered at face value but our recommendations for the use of CAC scoring in this statement assume that best practice standards are followed, including appropriate specialist follow‐up if indicated.

### Suggested approach to CAC measurement in practice

Taking the above information together, the Heart Foundation position is that CAC scoring can have a role in reclassification of absolute CVD risk for asymptomatic individuals. It is vital that any decision to use CAC scoring to reclassify risk status is informed by patient–clinician discussion of benefits, harms, preferences, cost and values.

Our suggested approach for incorporating our conditional recommendations for CAC scoring in primary prevention (Box 3) begins with risk assessment.

Box 3 **National Heart Foundation of Australia algorithm for coronary artery calcium (CAC) scoring in primary prevention of cardiovascular disease**

**Figure:**
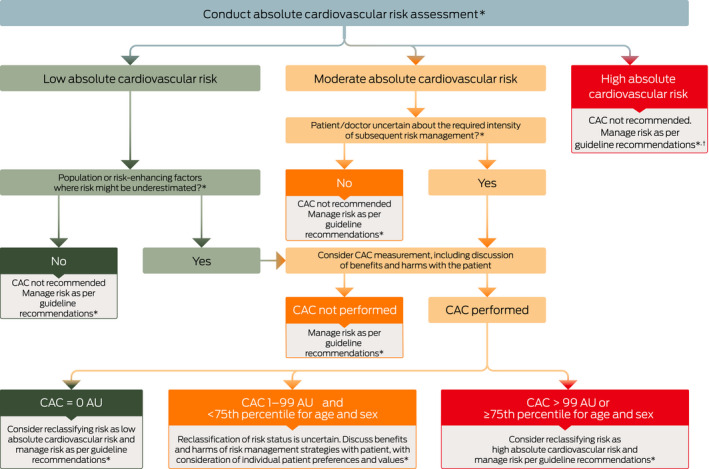
AU = Agatston units. * As assessed using the absolute cardiovascular disease risk calculator. † For the purpose of reclassifying risk. The use of calcium scoring to detect subclinical atherosclerosis may be considered in patients with familial hypercholesterolaemia (FH) in line with recent guidance from FH Australasia Network.^47^

Practice advice:

- Before consideration of CAC scoring, assess CVD risk on clinical grounds. If the person is aged over 45 years (or over 30 years if Aboriginal or Torres Strait Islander^48^), this should include an NVDPA absolute risk assessment.^3^
- The 2012 NVDPA absolute risk algorithm may underestimate risk in certain populations, such as Aboriginal and Torres Strait Islander peoples,^49^ or in others with known risk‐enhancing factors not fully captured in the NVDPA algorithm (Box 4). CAC scoring could be considered in some instances to reclassify risk.
- CAC scoring is not necessary in people already determined to be at high absolute cardiovascular risk, for the purpose of refining absolute risk assessment.^43^, ^44^
- CAC scoring could be considered in people with moderate risk for whom management intensity is uncertain. For example, when the initial risk status is close to the threshold for high risk status.
- A CAC score of 0 AU could reclassify a moderate risk person to low absolute risk status, although it does not rule out the presence of non‐calcified plaque. Apply caution in underestimating the risk in the presence of certain risk‐enhancing factors (eg, Aboriginal and Torres Strait Islander population, cigarette smoking, diabetes, and a family history of CVD^39^, ^40^, ^41^).
- For CAC scores of 0 AU, subsequent risk management strategies should follow contemporary guidelines for absolute CVD risk (eg, the 2012 NVDPA guidelines).^3^
- Where a CAC score is 1–99 AU and < 75th percentile for age and sex (Box 5), reclassification of risk status is uncertain.
- For CAC scores of 0 AU, an interval of 5 years is reasonable if considering a repeat CAC score, based on available evidence for conversion to positive CAC scores.^45^, ^46^
- CAC scores > 99 AU or ≥ 75th percentile for age and sex (Box 5) could reclassify a person indefinitely to high absolute risk status. Repeat CAC testing is not warranted in this group.
- For CAC scores > 99 AU or ≥ 75th percentile, subsequent risk management strategies, including the use of antihypertensive and lipid‐lowering therapies, should follow contemporary guidelines for management of absolute CVD risk; for example, the 2012 NVDPA absolute CVD risk guidelines^3^ and the 2016 Heart Foundation hypertension guidelines.^51^

Box 4 **Selected cardiovascular disease (CVD) risk‐enhancing factors from United States guidelines^41^, ^42^ that are not fully captured in the 2012 National Vascular Disease Prevention Alliance algorithm**

**Table:** 

Selected CVD risk‐enhancing factors
Family history of premature atherosclerotic CVD
Primary hypercholesterolaemia (LDL ≥ 4.1 mmol/L, non‐HDL ≥ 4.9 mmol/L)
Persistently elevated triglyceride levels (> 1.98 mmol/L)
Metabolic syndrome
History of premature menopause
History of pregnancy‐associated conditions that increase later atherosclerotic CVD risk (eg, preeclampsia)
Chronic inflammatory conditions (eg, rheumatoid arthritis)
High risk ethnicity (eg, south Asian populations, Aboriginal and Torres Strait Islander peoples)
Other lipids or biomarkers associated with increased atherosclerotic CVD risk, if measured: elevated high sensitivity C‐reactive protein level; elevated lipoprotein(a) level; elevated apolipoprotein B level; and reduced ankle‐brachial index (< 0.9)

LDL = low density lipoprotein; non‐HDL = non high density lipoprotein.

Box 5 Normal distribution of coronary artery calcium scores (AU) in a healthy cohort stratified by age and sex*

**Table:** 

Percentile	Men				Women			
	< 45 years	45–54 years	55–64 years	65–74 years	< 45 years	45–54 years	55–64 years	65–74 years
25th	0	0	0	40	0	0	0	0
50th	0	0	30	173	0	0	0	4
75th	0	21	162	585	0	0	17	43
90th	8	108	315	1230	0	1	91	212

AU = Agatston units; CAC = coronary artery calcium. * Source: adapted from Hoffmann.^50^

## Conclusion

The Heart Foundation’s position is that CAC scoring can have a role in reclassification of absolute cardiovascular risk for selected patients in Australia, in conjunction with traditional absolute risk assessment and as part of a shared decision‐making approach that considers the preferences and values of individual patients. We call for more research to define the role of CAC‐guided risk assessment and management in the Australian population.

## Competing interests

Warrick Bishop receives consultancy fees to report on coronary artery calcium scoring test results, and has written a book on the subject. He is paid membership fees for his website, which provides information about heart attack prevention. Clara Chow receives consultancy fees to report on coronary artery calcium scoring test results. Danny Liew receives honoraria to conduct cost‐effective analyses for pharmaceutical companies.

## Provenance

Not commissioned; externally peer reviewed.

## Supporting information

Supplementary Material Supplementary appendix, tables and figure
