# Supplementary material for: National Heart Foundation of Australia: position statement on coronary artery calcium scoring for the primary prevention of cardiovascular disease in Australia
Source: Med J Aust. 2021 May 7;214(9):434–9. doi: 10.5694/mja2.51039 (PMC8252756; doi:10.5694/mja2.51039)
Supplement: Supplementary file 1 — Supplementary Material Supplementary appendix, tables and figure [file MJA2-214-434-s001.pdf]

MJA
The Medical Journal of Australia

Supporting Information

Supplementary material

This appendix was part of the submitted manuscript and has been peer reviewed.
It is posted as supplied by the authors.

Appendix to: Jennings GLR, Audehm R, Bishop W, et al. National Heart Foundation Australia position statement: coronary artery calcium scoring for the primary prevention of cardiovascular disease in Australia. *Med J Aust* 2021; doi: 10.5694/mja2.51039.

Appendix 1 NHFA CAC scoring position statement: GRADE evidence appraisal

Preamble

The body of evidence for the use of coronary artery calcium (CAC) scoring in primary prevention of cardiovascular disease (CVD) was appraised for certainty to inform the National Heart Foundation of Australia (NHFA) Coronary artery calcium (CAC) scoring position statement. Studies relevant to CAC scoring from a 2018 systematic review of non-traditional risk factors in CVD risk assessment[1] were used as a foundation for this review. An additional literature search, hand-searching of reference lists from key guidelines and surveillance of key journals resulted in two additional studies being included for appraisal, using the inclusion criteria given in Table 1. Key outcomes were selected, and studies grouped accordingly. The certainty of evidence for each outcome was assessed after consideration of the five key domains in the GRADE approach for evidence appraisal (Figure 1).[2]

Challenges in this methodology arose from the differences in how the GRADE approach is applied to therapeutic research compared to prognostic research, especially the application to studies assessing prediction performance. The latter is an evolving area of methodology research, and clarification of the most reasonable approach was sought in literature, and via correspondence with the Adelaide GRADE centre.[3-5] Clarification was also sought for application of the GRADE approach when a body of evidence has been summarised narratively, rather than by pooled estimate of effect.[6] The strength of the recommendations, as detailed in the NHFA CAC position statement, was determined by expert consideration of: the certainty of the evidence; the benefits and harms of CAC scoring; patient preferences and values and resource considerations.

Table 1 Evidence appraisal inclusion criteria

**Population**	Asymptomatic patients without known cardiovascular disease (CVD).
**Intervention**	Coronary Artery Calcium score (to assess risk or guide treatment).
**Comparison**	Traditional/existing CVD risk assessment methods including Framingham Risk Score (FRS) and Pooled Cohort Equation (PCE).
**Outcomes**	Improvement in CVD risk prediction measures, reduction in CVD events.

Results:

The results of the GRADE evidence appraisal are summarised in Tables 2 and 3.

Several cohort studies reported varying improvements in calibration, discrimination, and reclassification when CAC is added to risk assessment models based on the Framingham Risk Score (FRS). Limitations in the ability to quantify the extent of improvement (by means of a pooled estimate effect) and concerns for applicability of the results to the Australian context contributed to a final rating of low certainty when using the GRADE approach (Table 3). Our evidence appraisal yielded results for the certainty of the evidence that are broadly consistent with international guidelines on management of blood cholesterol[7, 8], but the certainty level was further downgraded due to indirectness of much of the evidence to the Australian context as described in Table 3. We acknowledge that direct comparisons between our appraisal and that of international guidelines are difficult due to differences in the appraisal frameworks used.

The evidence of benefit from specific treatment guided by CAC results is of very low certainty with respect to reductions in CVD events. Two randomised, clinical trials did not find a difference in rates of CVD events when treatment was guided by CAC scoring and traditional risk assessment, compared to traditional risk assessment results alone (Table 2). One cohort analysis found that CAC was able to predict benefit from statin therapy in an asymptomatic population. Broadly, significant concerns for risk of bias and for the applicability of the results to the Australian context contributed to a final rating of very low certainty in these findings when using the GRADE approach (Table 3).

GRADE ratings of recommendations:

1. **CAC scoring may be considered for selected people with Intermediate absolute cardiovascular risk, as assessed by the NVDPA absolute cardiovascular risk algorithm, AND where decisions about the intensity of subsequent risk management will be influenced.**

   GRADE Evidence Certainty: *Low.* GRADE Recommendation Strength: *Conditional.*

2. **CAC scoring may be considered for selected people with Low absolute cardiovascular risk, as assessed by the NVDPA absolute cardiovascular risk algorithm, AND who have additional risk factors or enhancers that may result in underestimation of risk. .**

   GRADE Evidence Certainty: *Low.* GRADE Recommendation Strength: *Conditional.*

3. **If CAC scoring is undertaken, a CAC score = 0 Agatston Units (AU) could reclassify a person to a Low absolute cardiovascular risk status; with subsequent management to be informed by patient/clinician discussion and follow contemporary recommendations for Low absolute cardiovascular risk.**

   GRADE Evidence Certainty: *Very Low*. GRADE Recommendation Strength: *Conditional.*

4. **If CAC scoring is undertaken, a CAC score >99 Agatston units (AU) OR ≥75th percentile for age and gender could reclassify a person to a High absolute cardiovascular risk status; with subsequent management to be informed by patient/clinician discussion and follow contemporary recommendations for High absolute cardiovascular risk.**

   GRADE Evidence Certainty: *Very Low*. GRADE Recommendation Strength: *Conditional.*

Table 2 Summary of evidence appraisal findings. [a] Serious concerns of indirectness, borderline concerns of risk of bias, inconsistency, and publication bias. [b] Serious concerns of indirectness, borderline concerns of risk of bias, inconsistency, imprecision and publication bias. [c] Very serious concerns of indirectness, serious concerns of risk of bias and borderline concerns of imprecision. [d] Very serious concerns of risk of bias, serious concerns of indirectness and borderline concerns of imprecision. [e] Serious to very serious concerns of risk of bias, borderline concerns of indirectness.

Outcome	Studies	Effect	Certainty (GRADE)
Improvement of calibration with CAC + traditional risk assessment models, compared to traditional risk assessment models alone.	8[9-16]	Demonstrated 'goodness of fit' when CAC added to Framingham Risk Score (FRS) model and Pooled Cohort Equation (PCE) model.	**LOW[a]**
Improvement of discrimination with CAC + traditional risk assessment models, compared to traditional risk assessment models alone.	19[9-27]	Improved measures of discrimination with CAC + FRS/PCE compared to FRS/PCE alone.	**LOW[b]**
Positive overall net reclassification of risk with CAC + traditional risk assessment models, compared to traditional risk assessment models alone.	15[9-16, 20, 21, 23-26, 28]	Positive overall Net Reclassification Index results for CAC + FRS/PCE compared to FRS/PCE alone.	**LOW[a]**
Improvement of CVD outcomes with CAC + traditional risk assessment algorithms compared to traditional risk assessment algorithms alone.	1[29]	No significant difference in CVD outcomes when CAC score added to FRS assessment and risk factor counselling, compared to FRS assessment and risk factor counselling in asymptomatic participants.	**VERY LOW[c]** (single study)
Improvement of CVD outcomes with CAC-guided statin therapy.	1[30]	No significant difference in CVD outcomes when treated with atorvastatin 20mg + Vitamin C + Vitamin E compared to placebo in asymptomatic patients with CAC score >= 80th percentile for age and gender	**VERY LOW[d]** (single study)
Ability of CAC score to predict benefit from statin therapy.	1[31]	Statin therapy was associated with a statistically significant reduced risk of MACE compared to no statin therapy in patients with CAC >0; but no significant difference in risk of MACE was found for statin therapy compared to no statin therapy in patients with CAC = 0.	**LOW[e]** (single study)

Table 3 GRADE evidence table

Outcome	Participants (studies)	Study Design	Findings	Certainty of the evidence (GRADE)	Rationale and Clinical Significance.
**Improvement of calibration with CAC + traditional risk assessment models, compared to traditional risk assessment models alone.**	29,775 (8)<br><br>Age ranges of cohorts studied:<br><br>45yr – 84yr (2 studies, n =12,620)<br><br>45yr – 75yr (2 studies, n = 8095)<br><br>>= 55yr (3 studies, n = 8114)<br><br>40yr – 65yr (1 study, N= 946)	Cohort analyses	Subjectively consistent findings of good model fit when CAC added to traditional risk assessment models, as measured by statistically non-significant Hosmer-Lemeshow chi-square and likelihood ratio chi-square test results. Limited data reported on the difference in calibration measure between CAC + traditional risk assessment models and traditional risk assessment models alone.	**LOW**<br><br>**Risk of bias:** Borderline concerns due to differences in calibration measure between intervention and comparator not always reported with a p value. One study with concerns for selection bias, one study demonstrated suspected data dredging.<br><br>**Inconsistency:** Borderline concerns due to subjectively consistent direction of findings but with a range of magnitude of calibration measure results.<br><br>**Imprecision:** Not able to be quantitatively assessed, but large number of participants is reassuring.<br><br>**Indirectness:** Serious concerns as many studies used calibrated versions of risk prediction models (FRS, PCE), with different risk strata that are not used in clinical practice for the study populations. Furthermore, baseline FRS and PCE risk prediction models are not used in Australia.<br><br>**Publication bias:** Not able to be quantitatively assessed but possibly present as only positive studies were found despite a comprehensive search strategy.	The initial evidence certainty level was considered High, as appropriate for cohort studies in prognostic research.[3] The evidence was downgraded one level for serious concerns of indirectness. The domains of risk of bias, inconsistency and publication bias were considered together as the effects were assessed as small and/or uncertain. Thus, the evidence certainty was downgraded a further level, resulting in final assessment as Low certainty.<br><br>The clinical significance of the findings is unclear due to observational study design, and the measure of calibration reported by the studies does not provide information on direction of miscalibration. A lack of summary statistic reporting and a lack of guidance for methods of pooled analysis of these performance measures makes meta-analysis unfeasible, resulting in inability to quantitatively assess inconsistency, imprecision and publication bias.

Outcome	Participants (studies)	Study Design	Findings	Certainty of the evidence (GRADE)	Rationale and Clinical Significance.
**Improvement of discrimination with CAC + traditional risk assessment models, compared to traditional risk assessment models alone.**	69569 (19)<br><br>Age ranges of cohorts studied:<br><br>45yr – 84yr (5 studies, n =21, 896)<br><br>45yr – 75yr (3 studies, n = 11,203)<br><br>>= 55yr (3 studies, n = 8114)<br><br>40yr – 75yr (1 study, n = 5185)<br><br>40yr – 65yr (2 studies, n = 8328)<br><br>>45yr (1 study, n = 1029)<br><br>F>=40yr M>=35yr (1 study, n = 3486)<br><br><=79yr (1 study, n = 6739)<br><br><=80yr (1 study, n = 1286)<br><br>Unclear (1 study, n = 2303)	Cohort analyses	Subjectively consistent, statistically significant increases in C statistic (AUC of ROC analysis) in the range of 0.038 to 0.16 (representing small to large improvements based on USPTF classifications of change in AUC[1]), for CAC + FRS compared to FRS. A small range of increases (0.02 – 0.04) which were less statistically significant were reported for CAC + PCE compared to PCE alone.	**LOW**<br><br>**Risk of bias:** Borderline concerns due to lack of reporting of confidence intervals. One study suspicious for data dredging. One study with concerns for selection bias.<br><br>**Inconsistency:** Borderline concerns due to subjectively consistent direction of findings but with a range of magnitude of the difference between C statistic for CAC + FRS/PCE compared to C statistic for FRS/PCE alone<br><br>**Imprecision:** Borderline concerns as in the few studies that did report confidence intervals for C statistics, some CIs for C statistic of CAC + FRS/PCE overlapped with the C statistic for FRS/PCE alone despite the difference in C statistic reaching statistical significance.<br><br>**Indirectness:** Serious concerns as many studies used calibrated versions of risk prediction models (FRS, PCE), with different risk strata that are not used in practice for the study populations. Furthermore, baseline FRS and PCE risk prediction models are not used in Australia.<br><br>**Publication bias:** Not able to be quantitatively assessed but possibly present as only positive studies were found despite a comprehensive search strategy.	The initial evidence certainty level was considered High, as appropriate for cohort studies in prognostic research.[3] The evidence was downgraded one level for serious concerns of indirectness. The domains of risk of bias, inconsistency, imprecision and publication bias were considered together as the effects were assessed as small and/or uncertain. Thus, the evidence certainty was downgraded a further level, resulting in final assessment as Low certainty.<br><br>The clinical significance of the findings is unclear due to the lack of guidance in literature as to how to characterise magnitude of changes in a C statistic, although USPTF stipulated a practical classification in their systematic review[1], which is used here. Comparison is limited between studies due to differences in risk score thresholds, CVD outcome composites and population groups. A lack of summary statistic reporting and a lack of guidance for methods of pooled analysis of these performance measures makes meta-analysis unfeasible, resulting in inability to quantitatively assess inconsistency, imprecision and publication bias.

Outcome	Participants (studies)	Study Design	Findings	Certainty of the evidence (GRADE)	Rationale and Clinical Significance.
**Positive overall net reclassification of risk with CAC + traditional risk assessment models, compared to traditional risk assessment models alone.**	57409 (15)<br><br>Age ranges of cohorts studied:<br><br>45yr – 84yr (4 studies, n =20,450)<br><br>45yr – 75yr (3 studies, n = 11,203)<br><br>>= 55yr (3 studies, n = 8114)<br><br>40yr – 75yr (1 study, n = 5185)<br><br>40yr – 65yr (1 study, n = 946)<br><br>F>=40yr M>=35yr (1 study, n = 3486)<br><br><=79yr (1 study, n = 6739)<br><br><=80yr (1 study, n = 1286)	Cohort analyses	Consistently positive overall NRIs with a range of 0.11 to 0.55 were reported for CAC + FRS compared to FRS alone. The NRIs for subgroup analysis of participants in the Intermediate Risk FRS group were with higher, ranging from 0.21 to 0.659. For the subgroup analysis of participants in the Low Risk FRS group, NRI results ranged from 0.12 to 0.414.<br><br>When there was distinction between event NRI and non-event NRI (not all studies made this distinction), some non-event NRIs were negative and some of these negative, non-event NRIs reached statistical significance.<br><br>NRIs for CAC + PCE compared to PCE alone were assessed in three studies, with a range of 0.08 – 0.36.	**LOW**<br><br>**Risk of bias:** The method of NRI computation was not always clear. Reported NRIs often did not distinguish between event and non-event. One study suspicious for data dredging. One study with concerns for selection bias.<br><br>**Inconsistency:** Borderline concerns due to subjectively consistent direction of findings but with a range of magnitude of the overall NRIs reported.<br><br>**Imprecision:** Not able to be quantitatively assessed, but large number of participants is reassuring.<br><br>**Indirectness:** Many studies used calibrated versions of risk prediction models (FRS, PCE), with different risk strata that are not used in practice for the study populations. Furthermore, baseline FRS and PCE risk prediction models are not used in Australia.<br><br>**Publication Bias:** Not able to be quantitatively assessed but possibly present as only positive studies were found despite a comprehensive search strategy.	The initial evidence certainty level was considered High, as appropriate for cohort studies in prognostic research.[3] The evidence was downgraded one level for serious concerns of indirectness. The domains of risk of bias, inconsistency and publication bias were considered together as the effects were assessed as small and/or uncertain. Thus, the evidence certainty was downgraded a further level, resulting in final assessment as Low certainty.<br><br>The overall NRI in most studies appear to be driven by event NRIs being larger than non-event NRIs, some of which were negative. The clinical implication of negative non-event NRIs is that some patients were being incorrectly reclassified upwards into a higher risk category and received statin therapy for no benefit. However, it is difficult to determine the appropriateness of reclassification for subjects that do not experience an event during a finite study period.<br><br>Comparison is limited between studies due to differences in risk score thresholds, CVD outcome composites and population groups. A lack of summary statistic reporting and a lack of guidance for methods of pooled analysis of these performance measures makes meta-analysis unfeasible, resulting in inability to quantitatively assess inconsistency, imprecision and publication bias.

Outcome	Participants (studies)	Study Design	Findings	Certainty of the evidence (GRADE)	Rationale and Clinical Significance.
**Improvement of CVD outcomes with CAC + traditional risk assessment models compared to traditional risk assessment models alone.**	2137 (1) EINSER Study (secondary outcome)<br><br>Age range: <=80yr<br><br>Mean: 58.5yr (+/- 8.4yr)	Randomised clinical trial	No significant difference in combined myocardial infarction and mortality rates between subjects randomised to receive FRS assessment and risk factor counselling + CAC score; compared to subjects that only received FRS assessment and risk factor counselling (2.1% v 1% respectively, p = 0.08).	**VERY LOW**<br><br>**Risk of bias:** Serious concerns as the study randomised participants into treatment and control groups at a ratio of 2:1 and stated that this was done to encourage enrolment in the trial, as the CAC scan was offered to participants at no cost. Methods of allocation concealment and blinding were not reported. Study size and length of follow up were not powered to detect CVD events.<br><br>**Inconsistency:** Not able to be assessed as single study.<br><br>**Imprecision:** Borderline concerns due to low number of events detected (33). Quantitative assessment not possible due to absence of summary statistic reporting for secondary outcome.<br><br>**Indirectness:** Very serious concerns of the generalisability of the study population; a highly affluent, highly educated population recruited on premise of CAC scanning at no cost. Data was not collected on the extent and nature by which CAC score drove changes in health behaviour compared to control group. FRS assessment and risk factor counselling different to risk assessment model and counselling provided in the Australian population.<br><br>**Publication Bias:** Not able to be assessed as single study.	The initial study certainty level was considered High, as appropriate for a randomised, clinical trial.[2] The study was downgraded by three levels in total, due to borderline concerns of imprecision in addition to serious and very serious concerns of risk of bias and indirectness respectively. Thus, the final assessment of the study certainty level is Very Low.<br><br>The clinical significance of the results is uncertain, as the results are from a single study that was not powered to detect the outcome of interest for our clinical question. Additionally, the study did not collect data for the extent and nature by which the CAC scoring drove changes in health behaviour in the treatment group compared to the control group.

Outcome	Participants (studies)	Study Design	Findings	Certainty of the evidence (GRADE)	Rationale and Clinical Significance.
**Improvement of CVD outcomes with CAC-guided statin therapy.**	1005 (1)<br>St Francis Heart Study<br><br>Age range:<br>50yr – 70yr<br><br>Mean:<br>59yr<br>(+/- 6yr)	Placebo-controlled randomised clinical trial	In a sample of asymptomatic subjects with a CAC >= 80th percentile for age and gender, there was no significant difference in a composite of irreversible CVD events rate in subjects treated with atorvastatin 20mg/Vitamin C/Vitamin E compared to placebo (6.9% v 9.9% respectively, p = 0.08).<br><br>Post hoc analysis in subgroup of participants with CAC > 400 found a statistically significant reduction of CVD events rate with intervention compared to placebo (8.7% v 15% respectively, p = 0.046).	**VERY LOW**<br><br>**Risk of bias:** Very serious concerns of bias. The intervention (atorvastatin 20mg) could have been confounded by inclusion of Vitamins C and E that were not included in the placebo. Although unlikely to affect CVD event rates, the presence of a possible confounder in the intervention with little rationale is concerning. There was a large drop-out rate (18.4% of study cohort) and the study was under-powered for detection of CVD events. The low dose of atorvastatin used in the intervention group could have resulted in undertreatment. It was reported that 14% of the control group began taking a statin at the direction of their primary physician. Both treatment and control groups were prescribed aspirin, which could have lowered the overall event rate detected for the study sample.<br><br>**Inconsistency:** Not able to be assessed as single study.<br><br>**Imprecision:** Borderline concerns due to low number of events detected (85). Quantitative assessment not possible due to absence of summary statistic reporting for secondary outcome.<br><br>**Indirectness:** Serious concerns of indirectness. The use of aspirin in the study sample is a concern for indirectness, in addition to risk of bias, as aspirin is not recommended for primary prevention in Australian practice. Also, the CAC threshold of 80th percentile for age and gender is different to the threshold recommended in clinical guidelines used for the study population (75th percentile is recommended per the 2018 ACA/AHA Guidelines for management of blood cholesterol)[7].<br><br>**Publication Bias:** Not able to be assessed as single study.	The initial study certainty level was considered High, as appropriate for a randomised, clinical trial.[2] The study was downgraded by three levels in total, due to borderline concerns of imprecision in addition to serious and very serious concerns of indirectness and risk of bias respectively. Thus, the final assessment of the study certainty level is Very Low.<br><br>The clinical significance of the results is uncertain, as the results are from a single study that was not powered to detect the outcome of interest for our clinical question. Additionally, the study administered aspirin to all subjects, however, aspirin is not recommended for primary prevention in Australian clinical practice.

Outcome	Participants (studies)	Study Design	Findings	Certainty of the evidence (GRADE)	Rationale and Clinical Significance.
**Ability of CAC score to predict benefit from statin therapy.**	13644 (1)<br>Mitchel et al 2018.<br><br>Age range:<br>>=18yr<br><br>Mean:<br>49.6yr<br>(+/- 8.4yr)	Registry cohort analysis	In a cohort of asymptomatic patients, statin therapy in patients with CAC >0 was associated with a reduced 10-year MACE rate (asHR 0.76, 95% CI 0.60 – 0.95, p = 0.015) compared to no statin therapy. Statin therapy in patients with CAC = 0 was not associated with a change in 10-year MACE rate (asHR 1.00, 95% CI 0.79 – 1.27, p = 0.99) compared to no statin therapy.<br><br>When analysed by subdivisions of CAC score, the benefit of statin use on MACE risk was significantly related to CAC severity (p < 0.001 for interaction), with the most pronounced effect in the subgroup of subjects with CAC 101 – 400 (asHR 0.32, 95% CI 0.21 – 0.48, p < 0.0001).	**LOW**<br><br>**Risk of bias:** Subjects were not randomised to statin therapy, although inverse weighting techniques were used to reduce impact of confounding variables, as initially the subjects receiving statin therapy were more likely to have a higher CAC score and other risk factors. Concerns for bias remain as a range of statins and dose intensities were used, and it was not reported if the dose intensity differed for subjects with CAC = 0 v CAC>0. Additionally, outcome data was obtained from administrative claims data, carrying the risk of coding inaccuracies.<br><br>**Inconsistency:** Not able to be assessed as single study.<br><br>**Imprecision:** No concerns of imprecision due to the high number of events detected in the study (532) and confidence intervals for the positive CAC group that did not cross the threshold of no effect.<br><br>**Indirectness:** Borderline concerns of indirectness regarding the generalisability of the study population (71% male) and the subdivision of CAC only by score and not by percentile for age and gender.<br><br>**Publication Bias:** Not able to be assessed as single study.	The initial study certainty level was considered High, as appropriate for cohort studies in prognostic research.[3] The risk of bias was judged to be severe enough to warrant downgrading by one or possibly two levels. Including consideration of the borderline risk of indirectness, a judgement was made to downgrade the study by two levels overall. Thus, the final assessment of the study certainty level is Low.<br><br>This is a single study, of low certainty, that demonstrates the ability of CAC scoring to predict benefit from statin therapy in asymptomatic patients. To improve confidence in these results, the findings would need to be replicated in randomised trial designs with a range of populations, and meta-analysis of individual patient data needed to estimate the magnitude of effect[5].

Heart Foundation

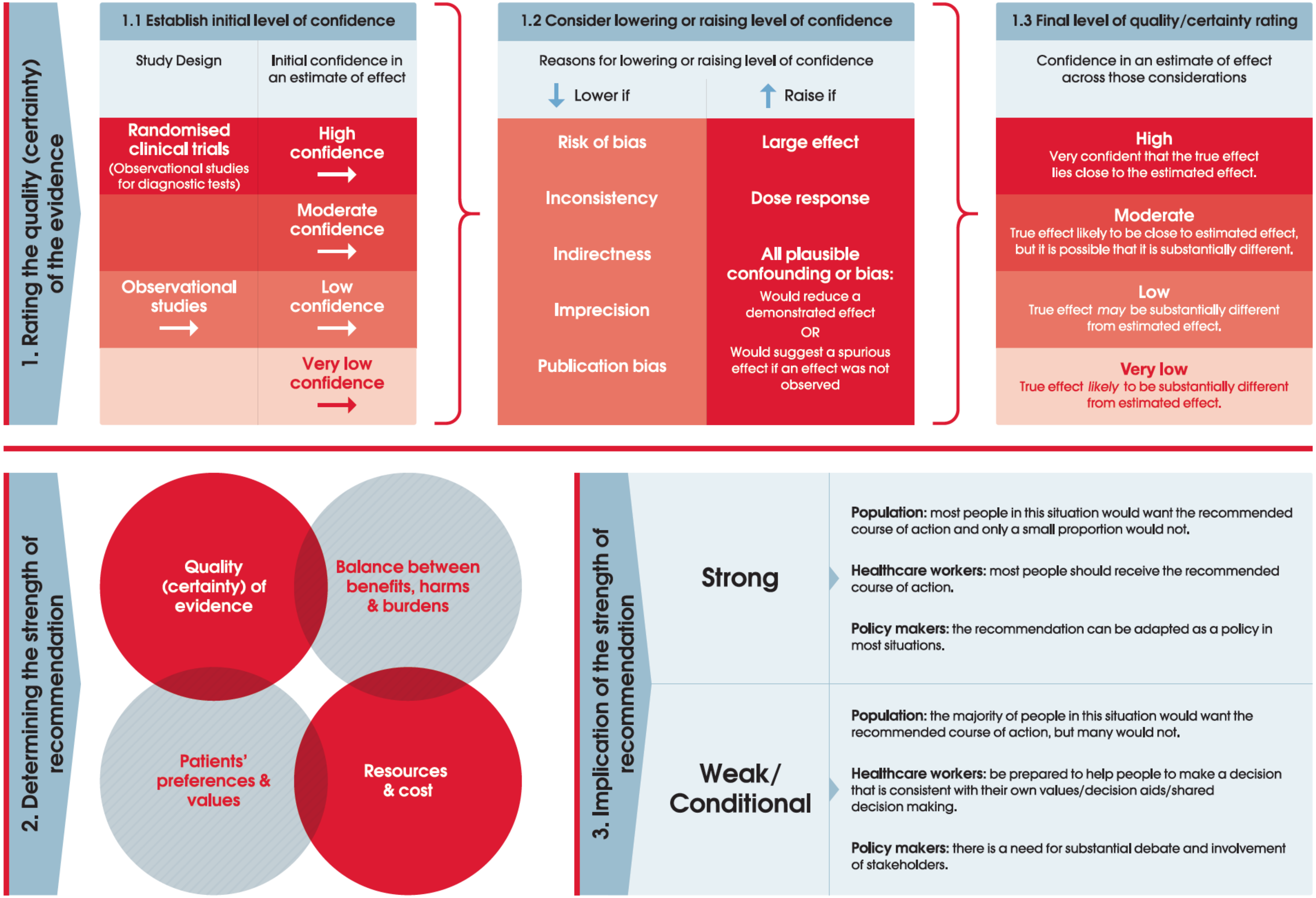

Figure 1 Summary of the GRADE approach for evidence appraisal and recommendation development when considering evidence for diagnostic tests.[2] *Adapted from the US GRADE Network with permission.*

References

1. Lin JS, Evans CV, Johnson E, et al. Nontraditional Risk Factors in Cardiovascular Disease Risk Assessment: Updated Evidence Report and Systematic Review for the US Preventive Services Task Force. *JAMA*. 2018;320(3):281-297.
2. Schunemann H, Brozek J, Guyatt G, et al, editors. GRADE handbook for grading quality of evidence and strength of recommendations [e-book] (updated Oct 2013). The GRADE Working Group; 2013. https://gdt.gradepro.org/app/handbook/handbook.html
3. Iorio A, Spencer FA, Falavigna M, et al. Use of GRADE for assessment of evidence about prognosis: rating confidence in estimates of event rates in broad categories of patients. *BMJ*. 2015;350:h870.
4. Steyerberg EW, Moons KGM, Van der Windt DA, et al. Prognosis Research Strategy (PROGRESS) 3: Prognostic Model Research. *PLoS Med*. 2013;10(2):e1001381.
5. Hingorani AD, Windt DAvd, Riley RD, et al. Prognosis research strategy (PROGRESS) 4: Stratified medicine research. *BMJ*. 2013;346:e5793.
6. Murad MH, Mustafa RA, Schünemann HJ, et al. Rating the certainty in evidence in the absence of a single estimate of effect. *Evid Based Med*. 2017;22(3):85-87.
7. Grundy SM, Stone NJ, Bailey AL, et al. 2018 AHA/ACC/AACVPR/AAPA/ABC/ACPM/ADA/AGS/APhA/ASPC/NLA/PCNA Guideline on the Management of Blood Cholesterol: A Report of the American College of Cardiology/American Heart Association Task Force on Clinical Practice Guidelines. *Circulation*. 2019;139:e1082–e1143.
8. Mach F, Baigent C, Catapano AL, Koskinas KC, Casula M, Badimon L, et al. 2019 ESC/EAS Guidelines for the management of dyslipidaemias: lipid modification to reduce cardiovascular risk: The Task Force for the management of dyslipidaemias of the European Society of Cardiology (ESC) and European Atherosclerosis Society (EAS). *Eur Heart J*. 2020 Jan 1;41(1):111-188.
9. Elias-Smale SE, Proença RV, Koller MT, et al. Coronary Calcium Score Improves Classification of Coronary Heart Disease Risk in the Elderly: The Rotterdam Study. *J Am Coll Cardiol*. 2010;56(17):1407-1414.
10. Erbel R, Mohlenkamp S, Moebus S, et al. Coronary risk stratification, discrimination, and reclassification improvement based on quantification of subclinical coronary atherosclerosis: the Heinz Nixdorf Recall study. *J Am Coll Cardiol*. 2010;56(17):1397-1406.
11. Polonsky TS, McClelland RL, Jorgensen NW, et al. Coronary artery calcium score and risk classification for coronary heart disease prediction. *JAMA.* 2010;303(16):1610-1616.
12. Möhlenkamp S, Lehmann N, Moebus S, et al. Quantification of Coronary Atherosclerosis and Inflammation to Predict Coronary Events and All-Cause Mortality. *J Am Coll Cardiol*. 2011;57(13):1455-1464.
13. Kavousi M, Elias-Smale S, Rutten JHW, et al. Evaluation of Newer Risk Markers for Coronary Heart Disease Risk Classification: A Cohort Study. *Ann Intern Med*. 2012;156(6):438-444.
14. Bos D, Leening MJ, Kavousi M, et al. Comparison of Atherosclerotic Calcification in Major Vessel Beds on the Risk of All-Cause and Cause-Specific Mortality: The Rotterdam Study. *Circ Cardiovasc Imaging*. 2015;8(12):e003843.
15. Chang SM, Nabi F, Xu J, et al. Value of CACS Compared With ETT and Myocardial Perfusion Imaging for Predicting Long-Term Cardiac Outcome in Asymptomatic and Symptomatic Patients at Low Risk for Coronary Disease: Clinical Implications in a Multimodality Imaging World. *JACC Cardiovasc Imaging*. 2015;8(2):134-144.
16. Fudim M, Zalawadiya S, Patel DK, et al. Data on coronary artery calcium score performance and cardiovascular risk reclassification across gender and ethnicities. *Data Brief*. 2016;6:578-581.
17. Greenland P, LaBree L, Azen SP, et al. Coronary artery calcium score combined with framingham score for risk prediction in asymptomatic individuals. *JAMA*. 2004;291(2):210-215.
18. Wong ND, Gransar H, Shaw L, et al. Thoracic aortic calcium versus coronary artery calcium for the prediction of coronary heart disease and cardiovascular disease events. *JACC Cardiovasc Imaging*. 2009;2(3):319-326.
19. Malik S, Budoff MJ, Katz R, et al. Impact of Subclinical Atherosclerosis on Cardiovascular Disease Events in Individuals With Metabolic Syndrome and Diabetes. The Multi-Ethnic Study of Atherosclerosis. *Diabetes Care*. 2011;34(10):2285-2290.
20. Rana JS, Gransar H, Wong ND, et al. Comparative value of coronary artery calcium and multiple blood biomarkers for prognostication of cardiovascular events. *Am J Cardiol*. 109(10):1449-1453.
21. Yeboah J, McClelland RL, Polonsky TS, et al. Comparison of novel risk markers for improvement in cardiovascular risk assessment in intermediate-risk individuals. *JAMA*. 2012;308(8):788-795.

22. Yeboah J, Erbel R, Delaney JC, et al. Development of a new diabetes risk prediction tool for incident coronary heart disease events: The Multi-Ethnic Study of Atherosclerosis and the Heinz Nixdorf Recall Study. *Atherosclerosis*. 2014;236(2):411-417.
23. Kavousi M, Desai CS, Ayers C, et al. Prevalence and prognostic implications of coronary artery calcification in low-risk women: A meta-analysis. *JAMA*. 2016;316(20):2126-2134.
24. Hoffmann U, Massaro JM, D'Agostino RB, Sr., et al. Cardiovascular Event Prediction and Risk Reclassification by Coronary, Aortic, and Valvular Calcification in the Framingham Heart Study. *J Am Heart Assoc*. 2016;5(2): e003144.
25. Yeboah J, Young R, McClelland RL, et al. Utility of Nontraditional Risk Markers in Atherosclerotic Cardiovascular Disease Risk Assessment. *J Am Coll Cardiol*. 2016;67(2):139-147.
26. Geisel MH, Bauer M, Hennig F, et al. Comparison of coronary artery calcification, carotid intima-media thickness and ankle-brachial index for predicting 10-year incident cardiovascular events in the general population. *Eur Heart J*. 2017;38(23):1815-1822.
27. Khera A, Budoff MJ, O'Donnell CJ, et al. The Astronaut Cardiovascular Health and Risk Modification (Astro-CHARM) Coronary Calcium Atherosclerotic Cardiovascular Disease Risk Calculator. *Circulation*. 2018;138:1819-1827.
28. Polak JF, Szklo M, O'Leary DH. Carotid Intima-Media Thickness Score, Positive Coronary Artery Calcium Score, and Incident Coronary Heart Disease: The Multi-Ethnic Study of Atherosclerosis. *J Am Heart Assoc*. 2017;6(1):e004612.
29. Rozanski A, Gransar H, Shaw LJ, et al. Impact of Coronary Artery Calcium Scanning on Coronary Risk Factors and Downstream Testing: The EISNER (Early Identification of Subclinical Atherosclerosis by Noninvasive Imaging Research) Prospective Randomized Trial. *J Am Coll Cardiol*. 2011;57(15):1622-1632.
30. Arad Y, Spadaro LA, Roth M, et al. Treatment of Asymptomatic Adults With Elevated Coronary Calcium Scores With Atorvastatin, Vitamin C, and Vitamin E: The St. Francis Heart Study Randomized Clinical Trial. *J Am Coll Cardiol*. 2005;46(1):166-172.
31. Mitchell JD, Fergestrom N, Gage BF, et al. Impact of Statins on Cardiovascular Outcomes Following Coronary Artery Calcium Scoring. *J Am Coll Cardiol*. 2018;72(25):3233-3242.
